# Clinical Challenges in the Management of Neuroendocrine Tumors

**DOI:** 10.3390/jcm10020257

**Published:** 2021-01-12

**Authors:** Francesco Panzuto

**Affiliations:** Digestive Diseases Unit, Sant’Andrea University Hospital, 00189 Rome, Italy; fpanzuto@ospedalesantandrea.it

Neuroendocrine tumors (NET) are rare and heterogeneous diseases, whose prognosis is affected by several factors including the primary tumor site, grading, somatostatin receptor expression, and disease staging [1]. Owing to these peculiar features, their management may be particularly difficult in terms of both the diagnostic work and the therapeutic approach. To address this topic, this Special Issue in the *Journal of Clinical Medicine* was dedicated to collecting high-quality scientific contributions focusing on potential clinical challenges in the management of patients with NETs.

Three studies investigated the prognosis and clinical outcomes in rare, specific subtypes of NETs, including large-cell neuroendocrine lung cancer [2], neuroendocrine breast cancer [3], and poorly differentiated bladder neuroendocrine carcinomas [4], providing useful data on the prognostic assessment of these diseases as well as for chemotherapeutic treatment, which remains the recommended therapeutic approach for patients with a poor prognosis.

Two studies focused on diagnostic work in relation to NETs: Özdirik et al. [5] suggested a potential diagnostic role for miR-29b serum levels as a novel biomarker for the diagnosis of NET, whereas Zhao et al. [6] summarized the procedures commonly used to visualize small insulinomas, with particular effort focused on the contribution of selective arterial calcium stimulation and hepatic venous sampling.

Several interesting recommendations are proposed in the review manuscripts presented in this Special Issue: (i) Pusceddu et al. [7] highlighted that diarrhea, although expected in the context of a small-bowel NET—particularly if associated with carcinoid syndrome—may have other etiologies, including previous surgery, concomitant medical treatments, and exocrine pancreatic insufficiency, which may develop in some patients receiving somatostatin analogs [8] or with mesenteric fibrosis. This last issue was intensely analyzed by Koumarianou [9] et al., who performed a systematic review of the literature concerning the pathogenesis and clinical management of mesenteric fibrosis, which may lead to severe symptoms, a deteriorated quality of life, and significant morbidity in patients with small-intestine NETs.

The NIKE study group, which includes several endocrinologists with significant expertise in NET management, provided a contribution to this Special Issue in the form of a detailed, comprehensive literature review on the potential role of peptide receptor radionuclide therapy (PRRT) in the setting of patients with medullary thyroid carcinoma [10], reporting a disease control rate of 62.4%, a figure that is in agreement with data from gastro-entero-pancreatic NETs. Peptide receptor radionuclide therapy was also the main topic covered by the study from del Olmo-García and co-workers, who investigated, through the robust methodology of a systematic review, the real risk of a hormonal crisis after PRRT in patients with syndromic functioning NETs or paraganglioma. Although this side effect is extremely rare, it needs to be carefully considered, requiring adequate monitoring and specific treatment, as detailed by the authors [11].

Beyond PRRT with 177-Lu-Dotatate—which has been approved worldwide for treating progressive, advanced, well-differentiated G1 and G2 gastro-entero-pancreatic NETs expressing somatostatin receptors—a number of clinical trials are ongoing that are investigating novel potential alpha-emitters or somatostatin antagonists in NETs. Additionally, there are more than 20 novel molecules (tyrosine kinase inhibitors or immunotherapeutic agents) under investigation in this setting of patients, which are described in the review titled “Emerging Treatment Options for Gastroenteropancreatic Neuroendocrine Tumors” by Cives et al. [12].

During the last year, owing to the global health emergency induced by the COVID-19 pandemic, the community of physicians dealing with NETs has been forced to adapt the management of these patients in accordance with the need to restrict movement, and requests to deeply revise the organization of most hospital departments. An initial report from the Italian Association for Neuroendocrine Tumors (www.ita-net.org) reported a significant impact of the COVID-19 pandemic on the number of newly diagnosed patients, as well as a reduction in surgical procedures for NET patients and delays in beginning scheduled PRRT [13]. A lower impact was reported in the study by Krug et al. [14] (published in this Special Issue), who observed that only a minority of physicians reported a major impact of the pandemic on care for NET patients in Germany, Austria, and Switzerland, suggesting different impacts of the COVID-19 pandemic among different areas and health systems, clearly depending on the incidence and dissemination of infections, which, unfortunately, are still increasing.

The data, recommendations, and scientific evidence collected for this Special Issue once again highlight that the management of patients with NETs still remains a challenge for physicians, given the rarity and the biological and clinical complexity of this disease, which is well known to necessitate a multidisciplinary team to achieve proper patient care [15].
